# Effects of an 18-week exercise programme started early during breast cancer treatment: a randomised controlled trial

**DOI:** 10.1186/s12916-015-0362-z

**Published:** 2015-06-08

**Authors:** Noémie Travier, Miranda J. Velthuis, Charlotte N. Steins Bisschop, Bram van den Buijs, Evelyn M. Monninkhof, Frank Backx, Maartje Los, Frans Erdkamp, Haiko J. Bloemendal, Carla Rodenhuis, Marnix A.J. de Roos, Marlies Verhaar, Daan ten Bokkel Huinink, Elsken van der Wall, Petra H.M. Peeters, Anne M. May

**Affiliations:** Julius Center for Health Sciences and Primary Care, University Medical Center Utrecht, PO Box 85500, STR 6.131, 3508 GA Utrecht, The Netherlands; Unit of Nutrition and Cancer, Catalan Institute of Oncology (ICO-IDIBELL), Barcelona, 08907 Spain; Department of Clinical Sciences, University of Barcelona, Barcelona, 08907 Spain; Comprehensive Cancer Organisation (IKNL), PO Box 19079, 3501 DB Utrecht, The Netherlands; Department of Rehabilitation, Nursing Sciences and Sport, University Medical Center Utrecht, Heidelberglaan 100, 3584 CG Utrecht, The Netherlands; Medical Oncology, St. Antonius Ziekenhuis, Koekoekslaan 1, 3435 CM Nieuwegein, The Netherlands; Internal Medicine – Medical Oncology, Obis Medisch Centrum, Dr vander Hoffplein 1, 6166 BG Sittard-Geleen, The Netherlands; Department of Internal Medicine, Medical Center, Maatweg 3, 3818 TZ Amersfoort, The Netherlands; Department of Radiation Oncology, University Medical Center Utrecht, Heidelberglaan 100, 3584 CG Utrecht, The Netherlands; Department of Surgery, Ziekenhuis Rivierenland, President Kennedylaan 1, 4002 WP Tiel, The Netherlands; Department of internal medicine, Hofpoort Ziekenhuis, Polanerbaan 2, 3447 GN Woerden, The Netherlands; Internal Medicine, Diakonessenhuis, Bosboomstraat 1, 3582 KE Utrecht, The Netherlands; Cancer Center, University Medical Center Utrecht, Heidelberglaan 100, 3584 CX Utrecht, The Netherlands

**Keywords:** Breast cancer, Exercise therapy, Fatigue, Randomised controlled trial

## Abstract

**Background:**

Exercise started shortly after breast cancer diagnosis might prevent or diminish fatigue complaints. The Physical Activity during Cancer Treatment (PACT) study was designed to primarily examine the effects of an 18-week exercise intervention, offered in the daily clinical practice setting and starting within 6 weeks after diagnosis, on preventing an increase in fatigue.

**Methods:**

This multi-centre controlled trial randomly assigned 204 breast cancer patients to usual care (n = 102) or supervised aerobic and resistance exercise (n = 102). By design, all patients received chemotherapy between baseline and 18 weeks. Fatigue (i.e., primary outcome at 18 weeks), quality of life, anxiety, depression, and physical fitness were measured at 18 and 36 weeks.

**Results:**

Intention-to-treat mixed linear model analyses showed that physical fatigue increased significantly less during cancer treatment in the intervention group compared to control (mean between-group differences at 18 weeks: −1.3; 95 % CI −2.5 to −0.1; effect size −0.30). Results for general fatigue were comparable but did not reach statistical significance (-1.0, 95%CI -2.1; 0.1; effect size -0.23). At 18 weeks, submaximal cardiorespiratory fitness and several muscle strength tests (leg extension and flexion) were significantly higher in the intervention group compared to control, whereas peak oxygen uptake did not differ between groups. At 36 weeks these differences were no longer statistically significant. Quality of life outcomes favoured the exercise group but were not significantly different between groups.

**Conclusions:**

A supervised 18-week exercise programme offered early in routine care during adjuvant breast cancer treatment showed positive effects on physical fatigue, submaximal cardiorespiratory fitness, and muscle strength. Exercise early during treatment of breast cancer can be recommended. At 36 weeks, these effects were no longer statistically significant. This might have been caused by the control participants’ high physical activity levels during follow-up.

**Trial registration:**

Current Controlled Trials ISRCTN43801571, Dutch Trial Register NTR2138. Trial registered on December 9th, 2009.

**Electronic supplementary material:**

The online version of this article (doi:10.1186/s12916-015-0362-z) contains supplementary material, which is available to authorized users.

## Background

Among the treatment-related side effects experienced by breast cancer patients, fatigue is the most often reported [1] and the most distressing [2]. Fatigue is reported by up to 30 % to 60 % of cancer patients during treatment and up to 25 % to 30 % still report fatigue many years after treatment [3]. Recent research indicates that exercise training during and after treatment may prevent and reduce cancer-related fatigue complaints [4–7]. A meta-analysis including studies evaluating exercise effects during adjuvant treatment for breast cancer found a small significant reduction of fatigue following exercise [8]. However, after excluding lower-quality studies that, for example, did not perform intention-to-treat analyses [9], the effect was no longer significant [8]. Recently, two new trials, not included in said meta-analysis, offering a 12-week supervised resistance exercise intervention to breast cancer patients, either during chemotherapy [10] or during radiotherapy [11], reported beneficial effects on fatigue. In both trials the control group received a progressive muscle relaxation intervention aiming at investigating the pure physiological exercise effect isolated from psychosocial effects. In contrast, in order to measure the effect in routine daily setting, we designed the Physical Activity during Cancer Treatment (PACT) trial relevant for facilitation of implementation of exercise training into clinical care [12].

In the PACT study, the effect of an 18-week aerobic and resistance exercise intervention was investigated. The intervention started as early as possible after breast cancer diagnosis and was offered at the patients’ treating hospital. The exercise training was supervised by physiotherapists working in daily clinical routine. The primary outcome was fatigue at 18 weeks. Furthermore, fatigue at 36 weeks and short- and long-term effects on secondary outcomes were assessed.

## Methods

### Setting and participants

The design of the two-arm randomised controlled PACT study has been published elsewhere [12]. In short, the present study was conducted in seven hospitals (one academic and six general hospitals) in the Netherlands between 2010 and 2013. Participants were invited by their clinician or oncological nurse during a regular outpatient clinic visit. The inclusion criteria were a definitive full histological breast cancer diagnosis <6 weeks before recruitment; stage M0 (i.e., no distant metastasis); scheduled for chemotherapy (as part of the treatment regime); aged 25 to 75 years; not treated for any cancer in the preceding 5 years (except basal skin cancer); able to read and understand the Dutch language; Karnovsky Performance Status of ≥60; and no contra-indications for physical activity. Inclusion was irrespective of the patients’ current physical activity level. The 6-week period was extended to 10 weeks if patients had a mastectomy with immediate reconstruction involving the use of tissue expander (n = 19). In the Netherlands, if indicated, patients usually receive radiotherapy for 3 to 4.5 weeks before chemotherapy if they are at low risk of distant metastases (less than four positive lymph nodes). Otherwise radiotherapy is scheduled after chemotherapy. By starting the intervention within 6 weeks post-diagnosis, we made sure that all patients participated in the 18-week exercise program during (part of their) chemotherapy.

The study was approved by the Medical Ethics Committee of the University Medical Centre Utrecht and the local Ethical Boards of the participating hospitals (i.e., St. Antonius Hospital, Nieuwegein; Diakonessenhuis Hospital, Utrecht; Meander Medical Centre, Amersfoort; Rivierenland Hospital, Tiel; Orbis Medical Centre, Sittard; Zuwe Hofpoort Hospital, Woerden).

Breast cancer patients willing to participate were asked to visit the study centre to confirm eligibility and sign informed consent. A concealed computer-generated randomisation, following a 1:1 ratio, stratified per age, adjuvant treatment (radiotherapy yes/no before chemotherapy), use of tissue expander, and hospital by sequential balancing, was used to allocate participants to study groups. Blinding of participants was not possible due to the nature of the study, but outcome measures were assessed by researchers not involved with the participants. Colon cancer patients were also included in the PACT study. Results for colon cancer patients will be presented separately to be able to address site-specific issues. Further, results of a formal cost-effectiveness analysis will be reported elsewhere.

### Intervention

An 18-week exercise programme was offered to patients randomised to the intervention group in addition to usual care. The programme included two aerobic and strength exercise sessions per week, supervised by a physiotherapist and incorporating cognitive behavioural principles of social Bandura’s cognitive theory [13]. The 60-min exercise classes included a warming-up (5 min), aerobic and muscle strength training (25 min each), and a cooling down (5 min) period. The exercise program was individualized to the patients’ preferences inventoried during the first exercise session and fitness level assessed by means of a cardiopulmonary exercise test and 1-repetition maximum muscle strength tests.

Intensity of the aerobic training was based on the heart rate at the ventilatory threshold as determined during baseline cardiopulmonary exercise test. The aerobic training included interval training of alternating intensity performed with a heart rate at (3 × 2 min increasing to 2 × 7 min) or below (3 × 4 min decreasing to 1 × 7 min) ventilatory threshold. Heart rate and the Borg scale of perceived exertion were monitored during the aerobic training.

Muscle strength training was performed for all major muscle groups: arms, legs, shoulder, and trunk. The training started with 2 × 10 repetitions (65 % one-repetition maximum) and gradually increased to reach 1 × 10 repetitions (75 % one-repetition maximum) and 1 × 20 repetitions (45 % one-repetition maximum) by the end of the programme. Training intensity was re-evaluated every four weeks by a submaximal cardiopulmonary exercise test and by repeating the 1-repetition maximum muscle strength tests. In addition, the participants of the intervention group were encouraged to be physically active for at least 30 min on at least three other days as recommended by the Dutch guidelines for physical activity [14]. This should include an aerobic component of moderate intensity in agreement with the participants’ fitness and desires.

Participants randomised to control received usual care and were asked to maintain their habitual physical activity pattern up to week 18. Then, they were allowed, for ethical reasons, to participate in exercise programmes, offered in the Netherlands to cancer patients after completion of primary treatment for over 10 years and are thus part of usual care.

### Outcome measures

Participants visited the study centre for outcome assessment at baseline, post-intervention (18 weeks), and after 36 weeks.

Fatigue, the primary outcome (at 18 weeks), was assessed using the Multidimensional Fatigue Inventory (MFI) and the Fatigue Quality List (FQL). The validated MFI is a 20-item questionnaire designed to measure general fatigue, physical fatigue, reduced activity, reduced motivation, and mental fatigue [15]. Scores range from 4 to 20, with higher scores indicating more fatigue. The FQL consists of 28 adjectives, clustered in four subscales: frustrating, exhausting, pleasant, and frightening, addressing the perception of fatigue [16]. Participants were asked to indicate the adjectives that fit their experienced fatigue.

Quality of life (QoL) was assessed using the validated 30-item European Organisation for Research and Treatment of Cancer Quality of Life Questionnaire C30 [17] and the 36-item Short Form Health Survey (SF-36) [18, 19]. Anxiety and depression were assessed using the validated Dutch language version of the 20-items Hospital Anxiety and Depression Scale [20].

Aerobic capacity was determined using a cardiopulmonary exercise test with continuous breathing gas analysis. After a 1-min warm-up at 20 W, cycling workload was increased every minute by a predetermined 10, 15, or 20 W until exhaustion or symptom limitation (dyspnoea and/or fatigue). Objective criteria for exhaustion were peak heart rate >85 % of age-predicted maximal HR, and respiratory exchange ratio >1.10. The load for each patient was defined according to the patient’s condition in order to reach exhaustion in about 10 min. The test was terminated on the basis of the patient’s symptoms or at the physician’s discretion. Peak oxygen uptake (VO_2peak_) was determined by taking the mean of VO_2_ values of the last 30 s before exhaustion. In addition, VO_2_ and power output were assessed at ventilatory threshold [21].

Thigh muscle strength was evaluated using a Cybex dynamometer at angular velocities of 60°/s and 180°/s. The highest peak torque of three repetitions was calculated for both velocities and both legs.

Handgrip strength was obtained taking the best score of two attempts provided by a mechanical handgrip dynamometer for both hands.

Body weight and height were measured to the nearest 0.5 kg and 0.5 cm, respectively, with patients wearing light clothes and no shoes.

Physical activity level was evaluated using the validated Short QUestionnaire to ASsess Health enhancing physical activity (SQUASH) [22]. This questionnaire contains questions on commuting activities, leisure-time and sports activities, household activities, and activities at work, and consists of three main queries: days per week, average time per day, and intensity referring to a normal week in the past months. We calculated the minutes per week of moderate to high intensity total physical activity and leisure and sport activity.

### Adherence

The attendance rate for the exercise sessions and the compliance with the protocol of the exercise sessions were recorded in a Case Record Form. Adherence to the exercise recommendation was recorded by the patients in an exercise log.

### Sample size calculation and statistical analysis

In order to detect a between-group change in fatigue of 2 units (±4 SD) at 18 weeks, corresponding to a medium effect size [23], we needed 75 participants in the intervention and control group (alpha = 0.05, power = 0.80) anticipating a drop-out of 10 %. With the current number of participants (n = 204) we are even able to detect smaller effect sizes.

Intention-to-treat mixed linear regression models were used to model the different outcome measures at 18 and 36 weeks. These models were adjusted for baseline values of the outcome, hospital, age, adjuvant radiotherapy, use of tissue expander, and tumour receptor status (triple negative/Her2Neu+, ER+ or PR+/Her2Neu+, ER– and PR–/Her2Neu–, ER+ or PR+). Between-group effects were modelled using outcome measurements obtained at 18 and/or 36 weeks; participants with only baseline data were not included in this analysis.

Within-group changes were modelled using outcome measurements obtained at the three time points (i.e., at baseline, and at 18 and/or 36 weeks) so all patients with at least one measurement were included in this analysis.

A sensitivity analysis was performed to assess whether having started chemotherapy before randomisation modified the intervention effect on fatigue. Standardized effect sizes (ES) were calculated by dividing the adjusted between-group difference of the post-intervention means by the pooled baseline standard deviation. According to Cohen, effect sizes <0.2 indicate ‘no difference’, effect sizes of 0.2 to 0.5 indicate ‘small differences’, effect sizes of 0.5 to 0.8 indicate ‘moderate differences’, and effect sizes ≥0.8 indicate ‘considerable differences’ [23].

We performed per-protocol analyses among adherent participants, i.e., excluding intervention and control participants reporting physical activity levels, respectively, below or above the 210 min of moderate-to-vigorous physical activity per week as assessed by the SQUASH questionnaire expected from participation in the intervention.

## Results

### Participants

**Fig. 1:**
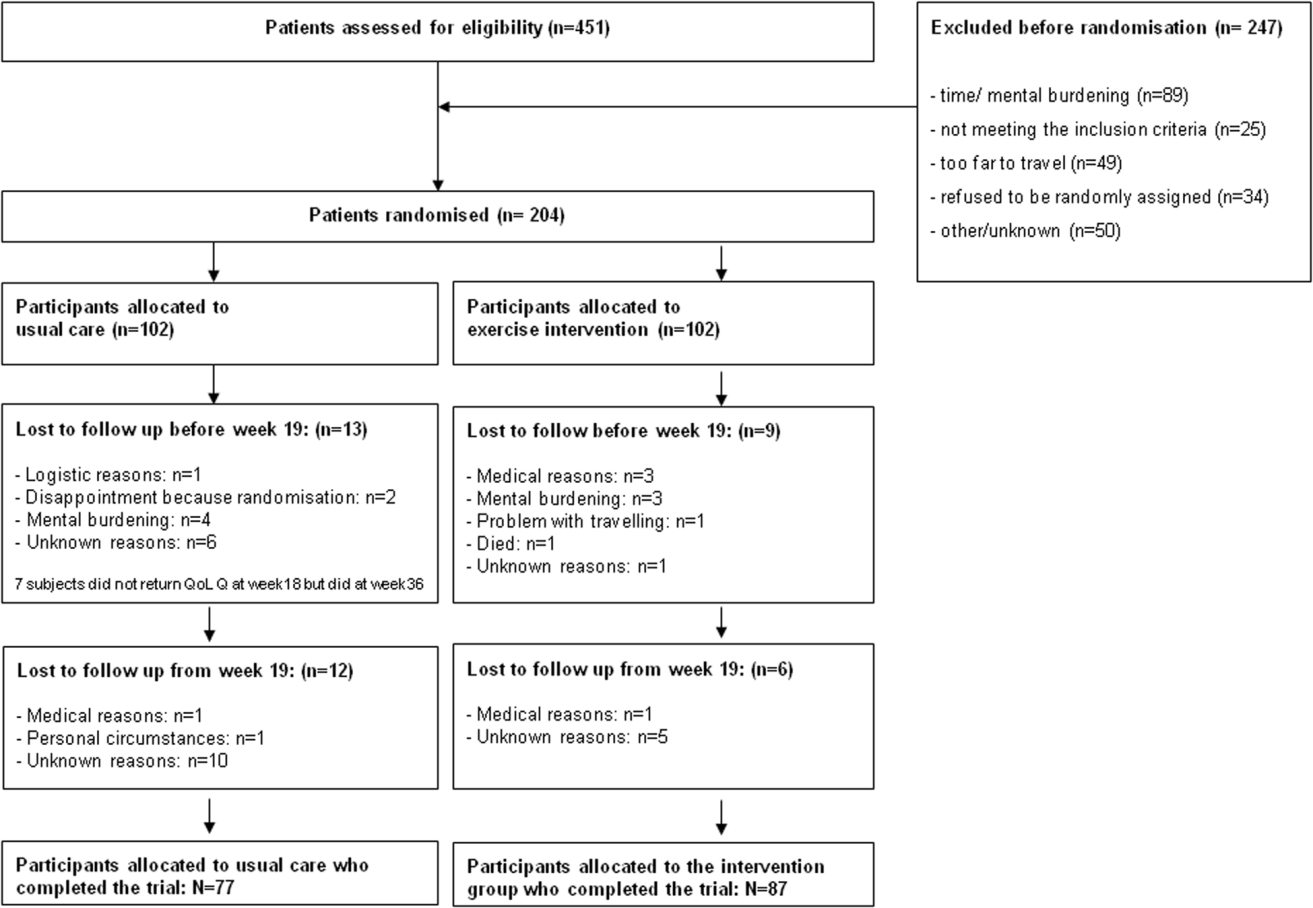
Flow chart of the PACT randomised clinical trial

Between January 2010 and December 2012, 451 breast cancer patients were invited to participate in the study (Fig. 1); 204 signed informed consent. The reasons for non-participation (n = 247) were ineligibility (n = 25), time/mental burden (n = 89), travel distance to hospital (n = 49), problem with random assignment (n = 34), or unknown (n = 50).

Overall, 15 of 102 participants allocated to the intervention group and 25 of 102 control group participants were lost to follow-up during the 36-week study period. In general, participants who did not complete the study (participants who did not come for outcome assessment at week 36) were at baseline significantly heavier, more fatigued, and reported more anxiety (results not shown).

At baseline, participants in the intervention and usual care group were comparable on most characteristics (Table 1) except that more women in the intervention group were highly educated (46.1 % vs. 35.3 %, respectively), had triple negative breast cancer (23.5 % vs. 11.8 %), and were post-menopausal (44.1 % vs. 32.4 %). Total physical activity levels (including activity at work) tended to be higher in the control group, whereas moderate-to-high leisure and sport physical activity levels were similar in both groups.

**Table 1:** Baseline characteristics of the participants of the PACT study

	Intervention group		Usual care group	
	n	%	n	%
**Education**				
Low	4	3.9	18	17.6
Medium	48	47.1	42	41.2
High	47	46.1	36	35.3
Unknown	3	2.9	6	5.9
**Marital status**				
Couple	79	77.5	76	74.5
Single	20	19.6	20	19.6
Unknown	3	2.9	6	5.9
**Menopausal status**				
Premenopausal	48	47.1	67	65.7
Postmenopausal	45	44.1	33	32.4
Unknown	9	8.8	2	2.0
**Radiotherapy**				
No	30	29.4	33	32.4
Yes	72	70.6	69	67.6
**Tissue expander**				
No	93	91.2	92	90.2
Yes	9	8.8	10	9.8
**Her2, ER, and PR receptors**				
Triple negative	24	23.5	12	11.8
Her2Neu+, ER+, or PR+	11	10.8	18	17.6
Her2Neu+, ER–, and PR–	10	9.8	2	2.0
Her2Neu–, ER+, or PR+	57	55.9	70	68.6
**Adjuvant treatment started before recruitment**				
Chemotherapy	36	35.3	36	35.3
Radiotherapy	27	26.5	35	34.3
None	39	38.2	31	30.4
	**Mean**	**SD**	**Mean**	**SD**
Age (years)	49.7	8.2	49.5	7.9
Height (cm)	168.1	6.6	169.1	6.5
Weight (kg)	72.8	13.2	76.0	15.4
BMI (kg/m^2^)	25.8	4.4	26.6	5.2
	**Median**	**IQR**	**Median**	**IQR**
Moderate to high intensity total PA performed before diagnosis (min/week)^*^	485	240–975	600	300–1440
Moderate to high intensity leisure and sport PA performed before diagnosis (min/week)	180	50–375	173	60–330

*IQR* interquartile range, *PA* physical activity

^*^ Including work, leisure, and sport activities

The PACT study with a duration of 18 weeks and a start within 6 weeks after diagnosis coincided with (all or part of) chemotherapy treatment in all patients; 72 patients had already started chemotherapy at recruitment; 62 patients had not started chemotherapy yet, but had started radiotherapy; and 70 patients had not started any treatment yet, but would start with chemotherapy early during the intervention period. Neo-adjuvant chemotherapy was still rare, and was used in less than 5 % of PACT participants. Treatment status at baseline was balanced between groups (Table 1).

### Adherence

Patients in the intervention group showed good adherence to the exercise programme: they participated in 83 % (interquartile range, 69 %–91 %) of the classes offered. Patients reported to be physically active according to the Dutch guideline for physical activity in 11 (interquartile range, 6–14) of the 18 weeks.

### Main outcomes

#### Fatigue

From pre- to post-intervention, participants in both groups reported significant increases in fatigue (Table 2). The increase in physical fatigue was significantly lower in the intervention group compared to control (mean between-group difference: −1.3; 95 % CI, −2.5 to −0.1; ES = −0.30). Although increases in general and mental fatigue, and in reduced activity were generally lower in the intervention group, no significant between-group differences were found. Over the same period, participants of both groups rated, on average to a comparable extent, their fatigue as more frustrating and exhausting. At 36 weeks, women reported fatigue levels and fatigue-related feelings that were in general similar to those observed at baseline with the exception of mental fatigue in the usual care group, which was reported to be still higher (mean: 1.0; 95 % CI, 0.1 to 1.9). No significant differences between the exercise and the usual care groups were found. No interaction was found between group assignment and chemotherapy timing (*P* >0.05).

**Table 2:** Effect of exercise on fatigue based on an intention-to-treat analysis

			Baseline to 18 weeks (i.e., post intervention)					Baseline to 36 weeks				
		Baseline	Within-group difference		Between-group difference			Within-group difference		Between-group difference		
		Mean (SD)	Mean	[95 % CI]	Mean	[95 % CI]	ES	Mean	[95 % CI]	Mean	[95 % CI]	ES
**Multidimensional Fatigue Inventory**												
General fatigue	UC	10.6 (4.1)	2.3	[1.4 to 3.3]		Reference		0.7	[−0.3 to 1.6]		Reference	
	I	10.1 (4.3)	1.9	[1.0 to 2.8]	−1.0	[−2.1 to 0.1]	−0.23	0.3	[−0.7 to 1.2]	−0.9	[−2.1 to 0.3]	−0.22
Physical fatigue	UC	10.6 (4.1)	2.2	[1.3 to 3.2]		Reference		−0.4	[−1.4 to 0.6]		Reference	
	I	9.9 (4.3)	1.6	[0.7 to 2.5]	−1.3	[−2.5 to −0.1]	−0.30	−0.3	[−1.3 to 0.6]	−0.5	[−1.7 to 0.8]	−0.11
Mental fatigue	UC	10.2 (4.1)	1.5	[0.6 to 2.4]		Reference		1.0	[0.1 to 1.9]		Reference	
	I	9.8 (4.0)	1.6	[0.8 to 2.5]	0.0	[−1.2 to 1.2]	0.01	0.6	[−0.3 to 1.5]	−0.6	[−1.8 to 0.7]	−0.15
Reduced motivation	UC	8.7 (3.4)	−0.3	[−1.0 to 0.5]		Reference		−1.5	[−2.3 to −0.8]		Reference	
	I	8.0 (3.5)	−0.2	[−0.9 to 0.5]	−0.2	[−1.1 to 0.7]	−0.06	−0.6	[−1.3 to 0.1]	0.7	[−0.2 to 1.7]	0.21
Reduced activity	UC	10.5 (3.7)	1.2	[0.3 to 2.1]		Reference		−1.1	[−2.0 to −0.2]		Reference	
	I	10.3 (3.8)	1.0	[0.1 to 1.9]	−0.4	[−1.5 to 0.8]	−0.09	−1.5	[−2.3 to −0.6]	−0.4	[−1.6 to 0.7]	−0.11
**Fatigue Quality List**												
Frustrating	UC	22.8 (24.7)	9.8	[3.6 to 16.0]		Reference		2.8	[−3.6 to 9.1]		Reference	
	I	15.9 (19.2)	7.9	[1.9 to 13.8]	−7.6	[−15.5 to 0.3]	−0.34	6.0	[−0.1 to 12.1]	−2.2	[−10.4 to 5.9]	−0.10
Exhausting	UC	6.9 (16.6)	4.9	[0.7 to 9.2]		Reference		−2.4	[−6.7 to 2.0]		Reference	
	I	6.9 (15.4)	5.0	[0.9 to 9.0]	0.8	[−4.6 to 6.2]	0.05	−0.7	[−4.9 to 3.5]	2.5	[−3.1 to 8.1]	0.16
Pleasant	UC	26.3 (20.7)	−4.6	[−9.6 to 0.3]		Reference		3.0	[−2.1 to 8.1]		Reference	
	I	29.2 (20.6)	−4.3	[−9.1 to 0.4]	2.6	[−3.2 to 8.5]	0.13	1.5	[−3.4 to 6.3]	0.5	[−5.6 to 6.6]	0.02
Frightening	UC	8.8 (17.1)	−1.4	[−5.0 to 2.2]		Reference		−4.9	[−8.6 to −1.2]		Reference	
	I	12.5 (17.5)	−3.3	[−6.7 to 0.2]	0.2	[−3.7 to 4.2]	0.01	−3.9	[−7.4 to −0.3]	2.9	[−1.2 to 7.0]	0.17

*ES* effect size, *I* intervention group, *UC* usual care group

Except for the pleasant dimension of the Fatigue Quality List, negative ES for fatigue dimensions indicate effects in favour of the exercise intervention group

Between-group effects were assessed using mixed models including the measurements obtained at 18 and 36 weeks, adjusted for age, hospital, radiotherapy, use of tissue expander, receptor status and the value of the outcome variable at baseline

Within-group effects were assessed using mixed models including the measurements obtained at baseline, 18 and 36 weeks, adjusted for age, hospital, radiotherapy, use of tissue expander, and receptor status

Baseline results and within-group differences were based on participants having baseline measurements (102 participants in each group). Between-group differences were based on participants for whom measurements at 18 or 36 weeks were available (93 intervention and 89 usual care)

### QoL and anxiety and depression

At 18 weeks, all participants generally reported significant decreases in QoL and in physical, cognitive, and social functioning as well as an increase in depression score, but none of these effects differed significantly between groups (Table 3). At 36 weeks, participants’ QoL and role functioning in both groups had significantly increased compared to baseline while cognitive functioning had slightly decreased, but no significant differences were observed between groups. Using the SF-36 (Additional file 1: Table S1), a significant between-group difference was observed at 18 weeks for the item ‘change in health’ with a difference in favour of the intervention group (mean: 11.3; 95 % CI, 3.4 to 19.1; ES = 0.47). At 36 weeks, both groups report higher scores for mental health, and the improvement was significantly lower in the intervention group (mean: −4.0; 95 % CI, −7.8 to −0.1; ES = −0.26).

**Table 3:** Effect of exercise on quality of life, anxiety, and depression based on an intention-to-treat analysis

			Baseline to 18 weeks (i.e., post intervention)					Baseline to 36 weeks				
		Baseline	Within-group difference		Between-group difference			Within-group difference		Between-group difference		
		Mean (SD)	Mean	[95 % CI]	Mean	[95 % CI]	ES	Mean	[95 % CI]	Mean	[95 % CI]	ES
**EORTC questionnaire**												
Quality of life	UC	72.5 (19.4)	−4.5	[−8.8 to −0.3]		Reference		5.5	[1.2 to 9.9]		Reference	
	I	74.8 (20.4)	−4.4	[−8.4 to −0.4]	2.3	[−2.4 to 6.9]	0.11	4.2	[0.0 to 8.3]	0.7	[−4.1 to 5.5]	0.03
Physical functioning	UC	85.0 (14.3)	−5.8	[−9.1 to −2.5]		Reference		0.8	[−2.5 to 4.2]		Reference	
	I	85.3 (14.2)	−4.2	[−7.3 to −1.1]	2.2	[−2.1 to 6.6]	0.16	0.4	[−2.8 to 3.6]	0.0	[−4.5 to 4.5]	0.00
Role functioning	UC	69.1 (23.6)	−6.7	[−12.4 to −1.0]		Reference		6.1	[0.3 to 11.9]		Reference	
	I	69.8 (23.5)	−1.3	[−6.7 to 4.1]	5.9	[−1.1 to 12.9]	0.25	10.4	[4.8 to 15.9]	4.1	[−3.1 to 11.4]	0.18
Emotional functioning	UC	79.2 (17.9)	2.5	[−1.0 to 6.0]		Reference		4.3	[0.7 to 7.9]		Reference	
	I	80.9 (18.2)	0.9	[−2.3 to 4.2]	−0.6	[−5.3 to 4.1]	−0.03	0.4	[−3.0 to 3.7]	−3.0	[−7.8 to 1.9]	−0.16
Cognitive functioning	UC	81.7 (23.4)	−7.8	[−13.1 to −2.5]		Reference		−6.9	[−12.3 to −1.5]		Reference	
	I	78.8 (23.6)	−10.1	[−15.1 to −5.1]	−4.4	[−11.3 to 2.5]	−0.19	−7.0	[−12.1 to −1.8]	−2.0	[−9.2 to 5.1]	−0.09
Social functioning	UC	80.9 (22.2)	−9.0	[−14.2 to −3.7]		Reference		1.3	[−4.1 to 6.7]		Reference	
	I	80.6 (22.0)	−7.6	[−12.6 to −2.7]	1.0	[−5.3 to 7.3]	0.04	3.7	[−1.4 to 8.9]	1.3	[−5.1 to 7.8]	0.06
Fatigue	UC	33.4 (23.4)	9.0	[3.9 to 14.1]		Reference		−1.5	[−6.7 to 3.7]		Reference	
	I	32.2 (25.1)	7.4	[2.5 to 12.2]	−3.0	[−9.1 to 3.1]	−0.12	−3.9	[−8.8 to 1.1]	−2.6	[−8.9 to 3.7]	−0.11
Pain	UC	22.9 (25.4)	−0.4	[−5.7 to 4.8]		Reference		−0.6	[−6.0 to 4.8]		Reference	
	I	24.0 (23.2)	−3.1	[−8.1 to 1.8]	−3.0	[−9.4 to 3.5]	−0.12	−0.5	[−5.7 to 4.6]	−0.1	[−6.7 to 6.6]	0.00
**Hospital Anxiety and Depression Scale questionnaire**												
Depression	UC	2.4 (2.7)	1.0	[0.4 to 1.5]		Reference		0.0	[−0.6 to 0.6]		Reference	
	I	2.5 (3.1)	0.8	[0.3 to 1.3]	0.0	[−0.8 to 0.7]	−0.01	0.0	[−0.5 to 0.6]	0.2	[−0.6 to 0.9]	0.06
Anxiety	UC	4.2 (3.0)	−0.5	[−1.1 to 0.1]		Reference		−0.3	[−0.9 to 0.3]		Reference	
	I	4.4 (3.4)	−0.2	[−0.8 to 0.3]	0.2	[−0.6 to 1.0]	0.06	0.2	[−0.4 to 0.8]	0.5	[−0.4 to 1.3]	0.15

*ES* effect size, *I* intervention group, *UC* usual care group

Negative ES for fatigue, pain, anxiety, and depression and positive ES for quality of life, physical functioning, role functioning, emotional functioning, cognitive functioning and social functioning indicate effects in favour of the exercise intervention group

Between-group effects were assessed using mixed models including the measurements obtained at 18 and 36 weeks, adjusted for age, hospital, radiotherapy, use of tissue expander, receptor status and the value of the outcome variable at baseline

Within-group effects were assessed using mixed models including the measurements obtained at baseline, 18 and 36 weeks, adjusted for age, hospital, radiotherapy, use of tissue expander, and receptor status

Baseline results and within-group differences were based on participants having baseline measurements (102 participants in each group). Between-group differences were based on participants for whom measurements at 18 or 36 weeks were available (93 intervention and 89 usual care)

### Physical fitness and body weight

At 18 weeks no significant between-group differences in VO_2peak_ and peak power output were observed. For VO_2_ and power output at ventilatory threshold significant differences in favour of the intervention group of 0.1 L/min (95 % CI, 0.0 to 0.2; ES = 0.31) and 9.4 W (95 % CI, 0.5 to 18.3; ES = 0.29) were respectively observed (Table 4). At 36 weeks, aerobic capacity did not differ between groups.

**Table 4:** Effect of exercise on aerobic capacity and muscle strength based on an intention-to-treat analysis

			Baseline to 18 weeks (i.e., post intervention)					Baseline to 36 weeks				
		Baseline	Within-group difference		Between-group difference			Within-group difference		Between-group difference		
		Mean (SD)	Mean	[95 % CI]	Mean	[95 % CI]	ES	Mean	[95 % CI]	Mean	[95 % CI]	ES
**Aerobic capacity**												
Peak VO_2_ (L/min)	UC	1.8 (0.3)	−0.2	[−0.3 to −0.1]		Reference		0.0	[0.0 to 0.1]		Reference	
	I	1.7 (0.4)	−0.2	[−0.2 to −0.1]	0.0	[−0.1 to 0.1]	0.06	0.0	[−0.1 to 0.1]	−0.1	[−0.2 to 0.0]	−0.20
Peak VO_2_/kg (mL/min/kg)	UC	23.8 (5.2)	−3.2	[−4.2 to −2.2]		Reference		0.3	[−0.6 to 1.3]		Reference	
	I	23.9 (5.6)	−2.8	[−3.7 to −2.0]	0.5	[−0.8 to 1.7]	0.09	−0.7	[−1.6 to 0.2]	−1.0	[−2.2 to 0.3]	−0.18
Peak power output (Watt)	UC	156.7 (34.3)	−19.5	[−24.4 to-14.6]		Reference		2.0	[−2.8 to 6.8]		Reference	
	I	152.3 (38.1)	−13.4	[−17.8 to −9.1]	4.0	[−2.3 to 10.4]	0.11	0.1	[−4.3 to 4.5]	−3.7	[−10.1 to 2.7]	−0.10
Peak heart rate (beat/min)	UC	169.9 (16.6)	−4.9	[−7.6 to −2.3]		Reference		−1.7	[−4.3 to 1.0]		Reference	
	I	167.6 (16.7)	−2.8	[−5.2 to −0.5]	0.9	[−2.7 to 4.5]	0.06	−2.2	[−4.7 to 0.2]	−1.6	[−5.3 to 2.0]	−0.10
VO_2_ at VT (L/min)	UC	1.2 (0.3)	−0.1	[−0.2 to 0.0]		Reference		0.1	[0.0 to 0.2]		Reference	
	I	1.2 (0.3)	0.0	[−0.1 to 0.0]	0.1	[0.0 to 0.2]	0.31	0.2	[0.1 to 0.2]	0.0	[−0.1 to 0.1]	0.12
Power output at VT (Watt)	UC	82.4 (33.8)	−11.5	[−18.4 to −4.6]		Reference		15.7	[8.7 to 22.7]		Reference	
	I	80.7 (31.4)	−2.7	[−8.9 to 3.6]	9.4	[0.5 to 18.3]	0.29	13.7	[7.4 to 20.1]	−2.0	[−11.0 to 7.0]	−0.06
**Muscle strength**												
Right knee extensor peak torque at 60°/s (Nm)	UC	106.0 (27.3)	−4.3	[−9.6 to 1.0]		Reference		−3.4	[−8.6 to 1.8]		Reference	
	I	102.6 (32.0)	4.4	[−0.5 to 9.2]	7.5	[0.9 to 14.2]	0.25	0.2	[−4.7 to 5.0]	2.7	[−3.8 to 9.3]	0.09
Right knee flexor peak torque at 60°/s (Nm)	UC	59.8 (22.7)	−3.6	[−8.2 to 1.0]		Reference		1.4	[−3.6 to 5.9]		Reference	
	I	58.6 (20.5)	7.8	[3.6 to 12.0]	9.1	[3.6 to 14.6]	0.42	8.5	[4.2 to 12.8]	5.8	[0.3 to 11.2]	0.27
Left knee extensor peak torque at 60°/s (Nm)	UC	97.7 (28.3)	−4.2	[−10.6 to 2.2]		Reference		−4.2	[−10.4 to 1.9]		Reference	
	I	96.4 (31.5)	6.7	[1.0 to 12.5]	9.9	[1.5 to 18.4]	0.33	2.7	[−3.1 to 8.5]	6.1	[−2.2 to 14.4]	0.20
Left knee flexor peak torque at 60°/s (Nm)	UC	61.3 (25.3)	−3.6	[−9.15 to 1.9]		Reference		−0.4	[−5.8 to 4.9]		Reference	
	I	59.3 (19.7)	8.3	[3.3 to 13.3]	10.1	[3.2 to 16.9]	0.45	4.6	[−0.5 to 9.6]	2.9	[−3.8 to 9.6]	0.13
Right knee extensor peak torque at 180°/s (Nm)	UC	58.0 (23.0)	−0.3	[−5.5 to 4.9]		Reference		1.9	[−3.2 to 6.9]		Reference	
	I	54.1 (22.4)	7.4	[2.7 to 12.1]	3.6	[−2.9 to 10.2]	0.16	7.4	[2.7 to 12.2]	1.5	[−4.9 to 8.0]	0.07
Right knee flexor peak torque at 180°/s (Nm)	UC	40.8 (20.6)	2.0	[−3.0 to 7.0]		Reference		3.7	[−1.1 to 8.6]		Reference	
	I	41.6 (20.3)	6.5	[2.0 to 11.1]	3.1	[−3.0 to 9.3]	0.15	6.7	[2.1 to 11.2]	2.2	[−3.9 to 8.2]	0.11
Left knee extensor peak torque at 180°/s (Nm)	UC	51.0 (21.9)	0.0	[−5.4 to 5.5]		Reference		−0.9	[−6.2 to 4.4]		Reference	
	I	49.1 (20.4)	5.1	[0.2 to 10.1]	3.9	[−3.2 to 10.9]	0.18	6.0	[1.1 to 11.0]	5.7	[−1.2 to 12.6]	0.27
Left knee flexor peak torque at 180°/s (Nm)	UC	39.1 (21.2)	3.4	[−1.1 to 7.9]		Reference		4.0	[−0.4 to 8.3]		Reference	
	I	40.5 (19.1)	5.4	[1.4 to 9.5]	1.2	[−4.4 to 6.8]	0.06	6.6	[2.5 to 10.6]	2.4	[−3.1 to 7.8]	0.12
Handgrip right (kgF)	UC	31.8 (5.7)	−0.9	[−1.9 to 0.1]		Reference		−0.7	[−1.7 to 0.4]		Reference	
	I	30.4 (5.8)	−0.2	[−1.1 to 0.7]	0.1	[−1.2 to 1.5]	0.02	0.1	[−0.8 to 1.0]	0.3	[−1.1 to 1.6]	0.05
Handgrip left (kgF)	UC	29.2 (6.0)	−0.8	[−2.0 to 0.3]		Reference		0.1	[−1.1 to 1.2]		Reference	
	I	27.8 (5.7)	0.2	[−0.8 to 1.2]	0.8	[−0.8 to 2.3]	0.13	0.6	[−0.4 to 1.6]	0.3	[−1.3 to 1.8]	0.04
**Body weight (kg)**	UC	76.0 (15.4)	1.5	[0.8 to 2.3]		Reference		1.6	[0.8 to 2.4]		Reference	
	I	72.8 (13.2)	1.9	[1.2 to 2.6]	0.1	[−0.9 to 1.2]	0.01	1.6	[0.9 to 2.3]	−0.1	[−1.2 to 0.9]	−0.01

*VT* ventilatory threshold, *ES* effect size, *I* intervention group, *UC* usual care group

Between-group effects were assessed using mixed models including the measurements obtained at 18 and 36 weeks, adjusted for age, hospital, radiotherapy, use of tissue expander, tumour receptor status, and the value of the outcome variable at baseline

Within-group effects were assessed using mixed models including the measurements obtained at baseline, 18 and 36 weeks, adjusted for age, hospital, radiotherapy, use of tissue expander, and tumour receptor status

Baseline results and within-group differences were based on participants having baseline measurements: body weight: 102 intervention (I) and 102 usual care (UC), aerobic capacity: 101 (I) and 98 (UC), leg strength: 78 (I) and 79 (UC), and hand grip: 98 (I) and 100 (UC)

Between-group differences were based on participants for whom measurements at 18 or 36 weeks were available: body weight: 90 (I) and 79 (UC), aerobic capacity: 88 (I) and 76 (UC), leg strength: 69 (I) and 64 (UC), and hand grip: 90 (I) and 79 (UC)

At 18 weeks, muscle strength in the intervention group was significantly higher for flexion and extension of both legs at 60°/s when compared to control (ES = 0.25–0.45; Table 4). No significant differences between groups were observed at 180°/s and for hand grip strength. Body weight at 18 and 36 weeks was similarly increased in both groups (Table 4).

### Per-protocol analyses

Overall, 89 % of intervention participants and 56 % of control participants reported being active with a moderate-to-high intensity for ≥210 min per week (120 min of supervised exercise and at least 30 min of unsupervised exercise on three other days). Per-protocol analyses showed, for both general and physical fatigue, moderate significant differences between participants, in both the exercise and the usual care group, who adhered to the protocol in favour of the intervention group with effect sizes of −0.54 and −0.77, respectively (Additional file 1: Table S2).

No serious adverse events related to exercise were observed during the study period.

## Discussion

The PACT study shows that an 18-week exercise intervention offered in routine clinical practice and starting shortly after breast cancer diagnosis has significant beneficial effects on physical fatigue, submaximal cardiorespiratory fitness, and muscle strength at 18 weeks compared to usual care. In the long-term, at 36 weeks, when adjuvant chemotherapy was completed, fatigue and fitness levels in both groups had returned to baseline levels. The intervention did not significantly affect QoL, anxiety, or depression.

The early start of our exercise intervention in breast cancer treatment coincided with adjuvant treatment. We therefore observed increased levels of fatigue at 18 weeks in both groups. However, the increase in the intervention group was significantly lower than for controls. The START trial, one large study comparable to our study, included 242 breast cancer patients receiving adjuvant treatment and showed changes in fatigue, QoL, anxiety, and depression that favoured the exercise intervention group but results were not statistically significant [24]. A study by Mutrie et al. [25] included 203 women in a 12-week supervised group exercise programme starting on average 6 months after diagnosis and also found non-significant beneficial effects of the intervention on fatigue and QoL. In contrast with these trials, we used a multi-dimensional fatigue scale and found a beneficial effect of the intervention on physical fatigue. Physical fatigue might be the fatigue dimension most sensitive to exercise. Indeed, Steindorf et al. [10] and Schmidt et al. [11], who compared the effects of resistance exercise and muscle relaxation on breast cancer patients’ fatigue during adjuvant therapy, also found beneficial effects especially on physical fatigue (ES = 0.3).

At 36 weeks, fatigue levels were back to baseline in both groups. This lack of difference might be explained by the fact that, from week 18, for ethical reasons, controls were allowed to participate in exercise programmes. Nevertheless, our results show long-term fatigue levels comparable across groups, and therefore it might be worth offering exercise interventions starting as early as possible after diagnosis to help breast cancer patients go through one of the most distressing periods of their life.

The non-significant changes in QoL corroborate the results of the START study [24]. The BEATE study also found no effect on QoL, but reported significant increases in role and social functioning after a resistance intervention offered during adjuvant chemotherapy [10]. These differences might be partly explained by the higher baseline scores observed in the present study or the exclusion of patients with baseline depression in the corresponding QoL analyses in the BEATE study.

The biological mechanisms that explain the beneficial effect of exercise on physical fatigue are not clear. Hypotheses include involvement in neurotoxicity of cancer treatments, chronic stress affecting the hypothalamic-pituitary-adrenal axis, systemic inflammatory responses, hormonal changes, reduced anaemia, or immune activation [2]. Skeletal muscles may act as an endocrine organ and induce myokine production associated with a reduced production and release of pro-inflammatory cytokines [26, 27]. Furthermore, while a self-perpetuating detraining state induces fatigue, physical training may break this vicious cycle [28]. Additionally, psychosocial mechanisms might play a role. Buffart et al. [29] showed that a supervised exercise program resulted in increased physical activity, general self-efficacy, and mastery in patients with cancer after treatment, which led to reduced fatigue and distress and consequently improved QoL.

The PACT exercise intervention also had beneficial effects on submaximal cardiorespiratory fitness and muscle strength, which corroborates findings from previous studies indicating that exercise during adjuvant treatment can prevent part of the deconditioning effect observed during cancer treatment [24, 30, 31]. Interestingly, in PACT, exercising during treatment did not only prevent losses but improved muscle strength. The significant results observed at submaximal level seem important since most daily activities are performed at submaximal level.

Compared to previous related studies [10, 11, 24, 25], the PACT study differed in the timing (i.e., early in the treatment process) and location (i.e., at the treating hospitals) of the intervention. In previous studies, the intervention was mostly delivered by the same physiotherapist(s) at a well-equipped research centre. In daily practice, however, the intervention will be given at different sites with different physiotherapists. The PACT study used this latter more pragmatic design. Although physiotherapists worked according to a standardized protocol the different locations may have added variability and reduced intervention effects. However, external generalizability is increased.

Strong features of the present study are the randomized design, the large sample size, and the high adherence to a supervised intervention offered in different clinical settings by different physiotherapists. Another feature resembling daily practice is that the intervention started within 6 weeks after diagnosis irrespective of the start of adjuvant treatment. Although no significant interaction was observed between group assignment and chemotherapy timing, treatment side-effects may have added variability to outcome measurements. The present study also has some limitations. Participants in the current study reported, on average, a high pre-diagnosis physical activity level and might thus not be the ones who needed the program most. The high level of physical activity reported by 56 % of the controls at 18 weeks may have led to an underestimation of the true effect. Indeed, per-protocol analyses showed that effects became stronger (e.g., ES = −0.77 for physical fatigue). However, per-protocol analyses should be interpreted with caution because of selective non-compliance. We offered a combined aerobic and strength exercise program to the patients. Therefore, we cannot distinguish what type of activity might have driven our results. Other limitations include the fact that physical activity was assessed by a questionnaire, as well as the relatively low participation rate and the lack of detailed information on patients who refused participation hampering generalization of results.

### Future directions

This study shows that exercise during adjuvant treatment of breast cancer is beneficial in reducing fatigue. Women with low physical activity levels might benefit more from exercise programs, although they might also be less interested in participating. Future studies should elucidate patients’ attitude, motivation, and barriers towards participation in exercise programs in order to specifically design exercise programs for the less active patients.

## Conclusions

The PACT trial shows that an exercise intervention offered in the daily clinical practice and starting early during adjuvant treatment is feasible and safe. The 18-week supervised exercise intervention reduces short-term physical fatigue and diminishment of cardiorespiratory fitness and improves muscle strength. At 36 weeks, effects were no longer statistically significant, probably due to participants’ high activity levels during follow-up. Exercise is beneficial during adjuvant breast cancer treatment by reducing the development of fatigue.

## Additional file

Additional file 1: Table S1. Effect of exercise on quality of life assessed using the SF-36 based on an intention-to-treat analysis. **Table S2.** Effect of the intervention on fatigue and quality of life taking into account compliance to study protocol.

## Abbreviations

ES: Effect size
FQL: Fatigue quality list
MFI: Multidimensional fatigue inventory
PACT: Physical activity during cancer treatment
QoL: Quality of life
SD: Standard deviation
SF-36: 36-Item short form health survey
SQUASH: Short questionnaire to assess health enhancing physical activity
VO_2peak_: Peak oxygen uptake
