# Supplementary material for: Effects of an 18-week exercise programme started early during breast cancer treatment: a randomised controlled trial
Source: BMC Med. 2015 Jun 8;13:121. doi: 10.1186/s12916-015-0362-z (PMC4461906; doi:10.1186/s12916-015-0362-z)
Supplement: Additional file 1: Table S1. — Effect of exercise on quality of life assessed using the SF-36 based on an intention-to-treat analysis. Table S2. Effect of the intervention on fatigue and quality of life taking into account compliance to study protocol. [file 12916_2015_362_MOESM1_ESM.docx]

**Supplemental table 1** Effect of exercise on quality of life assessed using the SF-36 based on an intention-to-treat analysis.

|  |  |  | **Baseline to 18 weeks (i.e., post intervention)** | | | | | **Baseline to 36 weeks** | | | | |
| --- | --- | --- | --- | --- | --- | --- | --- | --- | --- | --- | --- | --- |
|  |  |  | **Within-group**  **difference** | | **Between-group difference** | | | **Within-group**  **difference** | | **Between-group difference** | | |
|  |  | **Mean (SD)** | **Mean** | **[95% CI]** | **Mean** | **[95% CI]** | **ES** | **Mean** | **[95% CI]** | **Mean** | **[95% CI]** | **ES** |
| **SF-36 questionnaire** |  |  |  |  |  |  |  |  |  |  |  |  |
| Physical functioning | UC | 77.7 (16.3) | -8.2 | [-11.9; -4.6] |  | Reference |  | 2.9 | [ -0.9; 6.6] |  | Reference |  |
|  | I | 79.2 (15.4) | -4.8 | [ -8.3; -1.3] | 4.8 | [ -0.2; 9.8] | 0.30 | 2.6 | [ -1.0; 6.2] | 0.8 | [ -4.4; 5.9] | 0.05 |
| Social functioning | UC | 68.2 (21.4) | -4.8 | [ -10.0; 0.4] |  | Reference |  | 10.3 | [ 4.9; 15.6] |  | Reference |  |
|  | I | 69.4 (23.5) | -3.6 | [ -8.5; 1.4] | 1.9 | [ -4.4; 8.2] | 0.08 | 9.9 | [ 4.8; 15.1] | -0.1 | [ -6.7; 6.4] | -0.01 |
| Physical role limitations | UC | 18.8 (26.8) | 5.6 | [ -3.0; 14.2] |  | Reference |  | 22.6 | [ 13.8; 31.4] |  | Reference |  |
|  | I | 25.8 (31.4) | -2.3 | [-10.4; 6.0] | -3.2 | [-14.4; 8.0] | -0.11 | 26.3 | [ 17.8; 34.9] | 8.7 | [ -3.0; 20.3] | 0.30 |
| Emotional role limitation | UC | 61.7 (40.9) | 8.7 | [ -1.4; 18.7] |  | Reference |  | 14.1 | [ 3.8; 24.3] |  | Reference |  |
|  | I | 63.4 (42.6) | 10.6 | [ 1.0; 20.3] | 2.4 | [ -9.2; 14.1] | 0.06 | 12.1 | [ 2.2; 22.0] | 0.6 | [-11.5; 12.7] | 0.01 |
| Mental health | UC | 72.7 (14.9) | 3.7 | [ 1.0; 6.5] |  | Reference |  | 6.2 | [ 3.4; 9.1] |  | Reference |  |
|  | I | 73.6 (15.2) | 3.2 | [ 0.6; 5.8] | -0.5 | [ -4.3; 3.2] | -0.03 | 2.2 | [ -0.5; 4.9] | -4.0 | [ -7.8; -0.1] | -0.26 |
| Vitality | UC | 58.5 (17.7) | -5.2 | [ -9.0; -1.5] |  | Reference |  | 6.1 | [ 2.3; 9.9] |  | Reference |  |
|  | I | 62.5 (16.7) | -7.5 | [-11.1; -4.0] | -0.6 | [ -5.5; 4.2] | -0.04 | 2.0 | [ -1.7; 5.7] | -2.9 | [ -7.8; 2.1] | -0.17 |
| Bodily pain | UC | 63.8 (25.4) | 6.5 | [ 0.8; 12.3] |  | Reference |  | 14.2 | [ 8.3; 20.1] |  | Reference |  |
|  | I | 66.8 (23.8) | 6.4 | [ 0.9; 11.9] | 2.5 | [ -4.3; 9.3] | 0.10 | 8.5 | [ 2.8; 14.2] | -3.0 | [-10.1; 4.0] | -0.12 |
| Health perception | UC | 62.6 (17.6) | -2.5 | [ -6.2; 1.3] |  | Reference |  | 4.4 | [ 0.6; 8.2] |  | Reference |  |
|  | I | 65.7 (16.6) | -2.3 | [ -5.8; 1.3] | 1.8 | [ -3.1; 6.8] | 0.11 | 1.8 | [ -1.9; 5.5] | -1.0 | [ -6.1; 4.1] | -0.06 |
| Change in health | UC | 33.8 (23.8) | -15.8 | [-22.7; -8.9] |  | Reference |  | 9.6 | [ 2.6; 16.7] |  | Reference |  |
|  | I | 38.4 (23.9) | -9.9 | [-16.5; -3.2] | 11.3 | [ 3.4; 19.1] | 0.47 | 11.2 | [ 4.4; 18.0] | 6.6 | [ -1.5; 14.7] | 0.28 |

ES: Effect size. I: Intervention group. UC: Usual care group.

Positive ES for all dimensions of the SF-36 indicate effects in favour of the exercise intervention group.

Between-group effects were assessed using mixed models including the measurements obtained at 18 and 36 weeks adjusted for age, hospital, radiotherapy, use of tissue expander, receptor status and the value of the outcome variable at baseline.

Within-group effects were assessed using mixed models including the measurements obtained at baseline, 18 and 36 weeks adjusted for age, hospital, radiotherapy, use of tissue expander, receptor status and the value of the outcome variable at baseline.

Baseline results and within-group differences were based on participants having baseline measurements (102 participants in each group). Between-group differences were based on participants for whom measurements at 18 or 36 weeks were available (93 intervention and 89 usual care).

**Supplemental table 2** Effect of the intervention on fatigue and quality of life taking into account compliance to study protocol.

|  |  | **Intention-to-treat** | | |  | **Per protocol** | | |
| --- | --- | --- | --- | --- | --- | --- | --- | --- |
|  |  | **Between-group difference** | | |  | **Between-group difference** | | |
|  |  | **Mean** | **95%CI** | **ES** |  | **Mean** | **95%CI** | **ES** |
| **MFI General fatigue** | **Week 18** | -1.0 | [ -2.1; 0.1] | -0.23 |  | -2.3 | [ -3.9; -0.7] | -0.54 |
|  | **Week 36** | -0.9 | [ -2.1; 0.3] | -0.22 |  | -1.1 | [ -2.8; 0.7] | -0.25 |
| **MFI Physical fatigue** | **Week 18** | -1.3 | [ -2.5; -0.1] | -0.30 |  | -3.3 | [ -5.0; -1.6] | -0.77 |
|  | **Week 36** | -0.5 | [ -1.7; 0.8] | -0.11 |  | -0.7 | [ -2.5; 1.2] | -0.15 |
| **MFI Mental fatigue** | **Week 18** | 0.0 | [ -1.2; 1.2] | 0.01 |  | -2.0 | [ -3.6; -0.4] | -0.49 |
|  | **Week 36** | -0.6 | [ -1.8; 0.7] | -0.15 |  | -1.4 | [ -3.1; 0.4] | -0.33 |
| **MFI Reduced motivation** | **Week 18** | -0.2 | [ -1.1; 0.7] | -0.06 |  | -0.4 | [ -1.7; 0.8] | -0.13 |
|  | **Week 36** | 0.7 | [ -0.2; 1.7] | 0.21 |  | 1.3 | [ -0.1; 2.6] | 0.38 |
| **MFI Reduced Activity** | **Week 18** | -0.4 | [ -1.5; 0.8] | -0.09 |  | -2.6 | [ -4.2; -1.1] | -0.72 |
|  | **Week 36** | -0.4 | [ -1.6; 0.7] | -0.11 |  | -0.9 | [ -2.5; 0.8] | -0.24 |
|  |  |  |  |  |  |  |  |  |
| **EORTC Quality of life** | **Week 18** | 2.3 | [ -2.4; 6.9] | 0.11 |  | 7.4 | [ 1.2; 13.7] | 0.37 |
|  | **Week 36** | 0.7 | [ -4.1; 5.5] | 0.03 |  | 0.8 | [ -6.0; 7.5] | 0.04 |
| **EORTC Physical functioning** | **Week 18** | 2.2 | [ -2.1; 6.6] | 0.16 |  | 9.0 | [ 3.3; 14.7] | 0.60 |
|  | **Week 36** | 0.0 | [ -4.5; 4.5] | 0.00 |  | -1.2 | [ -7.2; 4.9] | -0.08 |
| **EORTC Role functioning** | **Week 18** | 5.9 | [ -1.1; 12.9] | 0.25 |  | 17.4 | [ 8.0; 26.9] | 0.72 |
|  | **Week 36** | 4.1 | [ -3.1; 11.4] | 0.18 |  | 5.5 | [ -4.6; 15.6] | 0.23 |
| **EORTC Emotional functioning** | **Week 18** | -0.6 | [ -5.3; 4.1] | -0.03 |  | 2.9 | [ -3.0; 8.8] | 0.18 |
|  | **Week 36** | -3.0 | [ -7.8; 1.9] | -0.16 |  | -2.3 | [ -8.6; 4.0] | -0.14 |
| **EORTC Cognitive functioning** | **Week 18** | -4.4 | [-11.3; 2.5] | -0.19 |  | 2.2 | [ -7.4; 11.8] | 0.10 |
|  | **Week 36** | -2.0 | [ -9.2; 5.1] | -0.09 |  | 4.2 | [ -6.2; 14.5] | 0.19 |
| **EORTC Social functioning** | **Week 18** | 1.0 | [ -5.3; 7.3] | 0.04 |  | 8.2 | [ -0.3; 16.6] | 0.37 |
|  | **Week 36** | 1.3 | [ -5.1; 7.8] | 0.06 |  | 0.7 | [ -8.3; 9.8] | 0.03 |
| **EORTC Fatigue** | **Week 18** | -3.0 | [ -9.1; 3.1] | -0.12 |  | -8.4 | [-17.2; 0.4] | -0.33 |
|  | **Week 36** | -2.6 | [ -8.9; 3.7] | -0.11 |  | -5.1 | [-14.5; 4.4] | -0.20 |
| **EORTC Pain** | **Week 18** | -3.0 | [ -9.4; 3.5] | -0.12 |  | -5.0 | [-13.6; 3.7] | -0.21 |
|  | **Week 36** | -0.1 | [ -6.7; 6.6] | 0.00 |  | 0.7 | [ -8.6; 10.1] | 0.03 |

ES: Effect size, MFI: Multidimensional Fatigue Inventory, EORTC: European Organisation for Research and Treatment of Cancer Quality of Life Questionnaire-C30

Negative ES for fatigue and pain and positive ES for quality of life, physical functioning, role functioning, emotional functioning, cognitive functioning and social functioning indicate effects in favour of the exercise intervention group.

Analyses adjusted for age, hospital, radiotherapy, use of tissue expander, receptor status and the value of the outcome variable at baseline.

Intention-to-treat analyses were based on participants for whom measurements at 18 or 36 weeks were available (93 intervention and 89 usual care). After exclusion of intervention participants reporting <210 minutes of moderate to vigorous physical activity per week at 18 weeks and usual care participants reporting ≥210 minutes of moderate to vigorous physical activity per week at 18 weeks per protocol analyses were based on 74 intervention and 34 usual care.
